# Predictors of Adolescent Internalizing and Externalizing Mental Health Symptoms: A School-Based Study in Southern India

**DOI:** 10.3390/ijerph21040393

**Published:** 2024-03-24

**Authors:** Varalakshmi Chandra Sekaran, Vidya Prabhu, Lena Ashok, Brayal D’Souza, Surekha Devadasa Shetty, Ravichandran Nair

**Affiliations:** 1 Department of Health Policy, Prasanna School of Public Health, Manipal Academy of Higher Education, Manipal 576104, India; 2 Department of Social and Health Innovation, Prasanna School of Public Health, Manipal Academy of Higher Education, Manipal 576104, India; 3 Division of Anatomy, Department of Basic Medical Sciences, Manipal Academy of Higher Education, Manipal 576104, India

**Keywords:** adolescent behaviors, mental health, internalizing behaviors, externalizing behaviors, predictor

## Abstract

Introduction: Mental illnesses are one of the major contributors to the overall burden of disease among the young. We investigated the predictors of emotional and behavioral problems among in-school adolescents in the Indian context. Methods: Using stratified sampling, 1441 adolescents were recruited to participate in the study in Udupi taluk. The study instruments included a socio-demographic pro forma and the adolescent self-reporting version of the Strengths and Difficulties Questionnaire (SDQ) to assess the emotional and behavioral problems among them. We explored the predictors of total difficulties, as well as externalizing and internalizing problems and gender differences. SPSS version 25 was used to analyze the data. Descriptive statistics, a Chi-square test for associations, an independent *t*-test to explore the gender differences, correlation analysis, and backward stepwise logistic regression for the predictors were used. Results: The mean age of the participants was 15.31 ± 0.76. An almost equal percentage of male (49.6%) and female (50.4%) participants provided data. Abnormal scores were highest under conduct problems (8.5%), and the total difficulties reached 5.1%. The male participants had higher levels of conduct, hyperactivity, peer relationship, and externalizing problems the while the female participants experienced higher levels of emotional and internalizing problems. It was observed that there was a significant positive relationship between age and emotional problems, conduct problems, hyperactivity, peer problems, and total SDQ score. An older age predicted an abnormal total difficulties score and externalizing and internalizing behaviors, while the type of school predicted the total difficulties and internalizing behaviors. Conclusion: The age of the adolescent, their gender, and the type of school they attended emerged as predictors of the emotional and behavioral problems among them.

## 1. Introduction

Mental health is enshrined in the World Health Organization’s concept of health [1,2]. While mental health conditions are prevalent in all regions of the world, accounting for one in seven, i.e., 14%, of all mental health conditions, a staggering 75% of individuals living in low-income and resource-constrained countries are reported to be affected [3]. As per the National Mental Health Survey of India, which was conducted between 2015 and 2016, the reported prevalence of mental illness among adolescents aged between 13 and 17 years old is 7.3% [4]. Mental illnesses are one of the major contributors to the overall burden of disease among the young, with the number of cases on the rise [3,5,6].

India is home to about 253 million adolescents. Adolescence is a period of rapid transition, marked by pubertal, physiological, and behavioral changes. Emerging evidence from India reports a variety of psychiatric morbidities such as depression, anxiety, stress, and schizophrenia among adolescents. The prevalence of psychiatric disorders among children and adolescents aged between 5 and 14 was 2.6 to 35.6% [7], while 2 to 3% of the population suffer from serious or incapacitating mental problems [8]. According to a review conducted in 2019 based on Indian studies, the prevalence of depression ranges between 1.2% and 21% in clinic-based studies; 3% and 68% in school-based studies; and 0.1% and 6.94% in community studies [9]. The broad range of the reported point prevalence is attributed to methodological discrepancies among the studies. Singh, M. et al. [10] assessed the prevalence of depression and the factors associated among school-going adolescents aged 13–18 and showed that 40% of the adolescents were screened positive for major depressive disorder: mild depression constituted 29.7%, moderate depression 15.5%, severely moderate depression 3.7%, and severe depression 1.1%. Bansal, V. et al. [11] estimated the prevalence of depression among adolescents studying at public school is 18.4%. Research shows that up to half of all mental health conditions begin during adolescence, and this is a universal phenomenon [2,12,13]. Precursors to emotional and behavioral problems may include immediate influences including the family, parental warmth and control [14,15,16,17], and peer relationships [18,19]. Hostile parenting is considered a risk factor for emotional and behavioral problems, whereas consistent parenting is a protective factor [20]. There is emerging evidence citing gender differences as well in the manifestation of behavioral problems, with a male preponderance towards externalizing behaviors and a female preponderance towards internalizing behaviors [21,22]. Behavioral problems are categorized into internalizing and externalizing symptoms. Internalizing symptoms are those experienced by the individual, including emotional and peer problems. Externalizing problems are behaviors aversive to others, such as behavioral problems, conduct problems, hyperactivity problems, etc. [23]. Neglecting the issue of the rise in mental health and behavioral difficulties among adolescents approaching maturity may have long-lasting consequences not just for individuals but for society as a whole. This necessitates early detection and the referral of adolescents with behavioral difficulties in order to reduce the severity and duration of their disorders; else, it might add to the existing burden on the health system. Schools in India are mainly divided into three types, which includes government, private, and aided. Government schools are governed, funded, and administrated by the state or central government. Private schools are governed, funded, and administrated by private organizations. Aided schools are supported by the government but are managed by private organizations [24]. In the present study, we explored the predictors of total difficulties as well as externalizing and internalizing problems and gender differences in all three types of schools.

## 2. Materials and Methods

### 2.1. Approval

Ethics clearance was obtained from the Kasturba Medical College and Kasturba Hospital Institutional Ethics Committee, Manipal, India. Prior to the data collection, written informed consent was obtained from all participants.

Permission to conduct the study through the schools was obtained from the Deputy Director of Public Instructions (DDPI). Further, permission was also obtained from the block education officers of both the Udupi and Brahmavar blocks.

### 2.2. Study Setting

The study was conducted in Udupi taluk, Udupi district. There are a total of 462 schools in Udupi taluk, divided under two blocks, namely Udupi and Brahmavar, with 215 and 247 schools, respectively. Screening of adolescents aged 14–16 years old was performed among students from government, aided, and private high schools from Udupi taluk following parental consent and adolescent assent.

### 2.3. Study Design

A cross-sectional study design was adopted. To estimate a prevalence of mental health conditions of 12.5% [4] on screening adolescents with a 95% confidence level, 15% relative precision, and a 10% non-response rate, a minimum of 1328 students needed to be screened. Lists of the schools in both blocks were obtained from the DDPI’s office. Stratified sampling technique was adopted, and the schools were stratified into government, aided, and private schools, following which proportional sampling was performed to achieve a sample size of 1328 students. Schools were selected using simple random sampling. Following permission from the school authorities, data were collected from all the students in each classroom, as each class was considered a cluster. Each class averaged 50–60 students in size. Overall, 1441 students provided complete data from 37 schools, including 13 government, 12 aided, and 12 private schools from the two blocks. A screening question on their history of mental health conditions was included in the questionnaire. Adolescents with a previous diagnosis of psychiatric morbidity were excluded from the study. Adolescents identified with mental health conditions during the screening were referred for further evaluation as required.

### 2.4. Data Collection Instruments

These included a short socio-demographic pro forma that included questions on age, gender, type of school, area of residence, and socioeconomic status.

Screening of the high school students was carried out using the Strengths and Difficulties (SDQ) Questionnaire [25]. The tool has been used in several Indian settings. The questionnaire was translated into Kannada, the local language and is available on the website at [26].

The SDQ (adolescent version) is a brief, 25-item behavioral screening questionnaire. The questionnaire is scored on a 3-point scale (0–2), with “0” denoting “Not True” and “2” denoting “Certainly True”. The questionnaire is divided into five subscales—emotional symptoms, conduct problems, hyperactivity, peer relationship problems, and pro-social behavior. The total cutoff score for the normal category is 0–15; for the borderline category, it is 16–19; and for the abnormal category, it is more than 20–40. For emotional problems, the cutoff scores for the normal, borderline, and abnormal categories are 0–5, 6, and 7–10, respectively. For conduct problems, the cutoff scores for the normal, borderline, and abnormal categories are 0–3, 4, and 5–10, respectively. For hyperactivity, the cutoff scores for the normal, borderline, and abnormal categories are 0–5, 6, and 7–10, respectively. For peer problems, the cutoff scores for the normal, borderline, and abnormal categories are 0–3, 4–5, and 6–10, respectively. For prosocial behavior, the cutoff scores for the normal, borderline, and abnormal categories are 6–10, 5, and 0–4, respectively.

The English/Kannada versions of the tool were used per the preference of the participants. Subgroup analysis was performed to measure externalizing behaviors, which are a composite of conduct problems and the hyperactivity/inattention domain, and internalizing behaviors were measured as a composite of emotional symptoms and peer relationship problems.

### 2.5. Measurements

School-going adolescents between the ages of 14 and 16 years old were sampled. Prior to the data collection, the project staff approached the school authorities and sought permission and met the potential participants at the convenience of the school. Following this, the participant information sheet and parental consent forms were provided to the parents to provide informed consent. Following this, the school was visited on a predetermined day to obtain assent from those students whose parents had agreed to provide consent. The data collection was then completed in the classrooms at the convenience of the school. The questionnaire was self-administered. The field staff were present to clarify any doubts that the students had. Following the data collection, the data was cleaned and entered into a database at the project office.

### 2.6. Statistical Analysis

SPSS version 25 (IBM, Bangalore, India) was used to analyze the data. Descriptive analysis and backward stepwise logistic regression were performed to identify predictors. The Chi-square and Fisher’s exact tests were performed to compare the proportions, and significance was set at *p* < 0.05. An independent sample *t*-test was performed to assess the gender differences in relation to the domains of SDQ. Correlation between the age of the participants and the domains of the SDQ was also performed.

## 3. Results

A total of 1441 adolescents participated Table 1. A higher percentage of the adolescents hailed from rural areas (55.3%). Almost equal percentages of male (49.6%) and female (50.4%) participants provided data. The mean age of the participants was 15.31 ± 0.76 with 15.5% aged 14 years old, 37.2% aged 15 years old, and 47.3% aged 16 years old. About 38.2% studied at government schools, 24% were from aided schools, and 37.8% were from private schools. The majority of the participants belonged to the below-the-poverty-line socioeconomic category (55.9%).

Table 2 depicts the self-reported emotional and behavioral problems of the adolescents across the SDQ domains. The internal consistency of the SDQ total difficulty score was 0.662. On assessing the total difficulty score using the SDQ, it indicated that most adolescents scored in the normal category, while 12.7% scored in the borderline category and 5.1% in the abnormal category. It was observed that the majority of adolescents scored in the normal category, while 3.1% to 17.8% fell under the borderline category. The fewest abnormal scores were seen under prosocial behaviors (1.8%), while the most were under conduct problems (8.5%) (Table 2).

Externalizing behaviors were assessed [27], and 8.3% of adolescents scored in the “slightly raised” category, 3.5% in the “high” category, and 1.1% in the “very high” category. Similarly, internalizing behaviors were assessed, and 17.3% of adolescents scored in the “slightly raised” category, 6.4% in the “high” category, and 10.8% in the “very high” category.

Using the Chi-square test, the type of school was found to be significantly associated with the total difficulty score (*p* = 0.002) and highly significantly associated with internalizing behaviors (*p* < 0.0001). However, externalizing behaviors were not found to be associated with the type of school (*p* = 0.07). There were no significant associations found between urban and rural areas and the SDQ scores.

Table 3 depicts the findings of the independent sample *t*-test for gender differences in relation to each of the SDQ domains, as well as externalizing and internalizing behaviors. Emotional problems were found to be significantly higher among the female participants (mean = 3.51, SD = 2.07, t = 4.65, *p* < 0.0001). The male participants scored higher compared to the female participants in assessing for conduct problems (mean = 2.39, SD1.58, t = 2.28, *p* = 0.023), hyperactivity (mean = 3.20, SD = 1.58, t = 3.06, *p* = 0.002), and peer relationships (mean = 2.52, SD = 1.48, t = 2.53, *p* = 0.011). On testing for the mean difference in the prosocial scores, it was found that the female participants scored higher (mean = 8.59, SD = 1.45, t = 4.88, *p* < 0.0001). The male participants scored higher in terms of externalizing behaviors (mean = 5.59, SD = 2.80, t = 3.24, *p* = 0.001), while the female participants scored higher in terms of internalizing behaviors (mean = 5.83, SD = 2.02, t = 2.02, *p* = 0.043). In terms of the mean difference in the total SDQ scores, gender differences were observed; however, this was not a significant finding. These findings indicate that the male participants had higher levels of conduct, hyperactivity, peer relationship, and externalizing problems while the female participants experienced higher levels of emotional and internalizing problems.

### 3.1. Correlation Analysis

Pearson’s correlation was performed between the symptom scales, and it was found that there was a low positive correlation overall: emotional problems were significantly correlated with peer problems (r = 0.264, *p* < 0.001), conduct problems (r = 0.354, *p* < 0.001), and hyperactivity (r = 0.354, *p* < 0.001); peer problems were correlated with conduct problems (r = 0.340, *p* < 0.001), hyperactivity (r = 0.216, *p* < 0.001), and emotional problems (r = 0.264, *p* < 0.001); and conduct problems were corelated with hyperactivity (r = 0.305, *p* < 0.001). Pearson’s correlation was performed to assess the relationship between the ages of the participants and the domains of the SDQ. It was observed that there was a significant positive relationship between age and emotional problems (r = 0.09, *p* = 0.001), conduct problems (r = 0.17, *p* < 0.0001), hyperactivity (r = 0.15, *p* < 0.0001), peer problems (r = 0.06, *p* = 0.03), and total SDQ score (r = 0.16, *p* < 0.0001). The prosocial domain was not significantly associated (r = 0.003, *p* = 0.91).

### 3.2. Regression Analysis

Predictors of the outcomes were assessed using backward stepwise logistic regression. Univariate and multivariate analyses of the predictors of total difficulties and externalizing and internalizing behaviors were performed.

The total difficulties score in the SDQ was assessed for predictors and factors, and the age of the participants and the type of school. These were found to be significant in the univariate analysis. These were then adjusted using multivariate analysis. The predictors of the total difficulties that emerged again included both the age of the participants and the type of school. In comparison with the adolescents from private schools, those in aided schools had a higher likelihood of having a higher total difficulties score (AOR 1.65, 95% CI (1.18–2.31), *p* = 0.004). These findings are depicted in Table 4.

A subgroup analysis of the externalizing and internalizing behaviors among adolescents was performed. The gender of the participants emerged as a significant predictor of externalizing behaviors. Being a male adolescent increased the odds of reporting externalizing behaviors by 1.49 times (AOR 1.49, 95% CI (1.09–2.04), *p* = 0.013). On assessing the predictors of internalizing behaviors, the gender of the participant, the age of the participant, and the type of school emerged as significant predictors. Being a female adolescent increased the odds of reporting internalizing behaviors by 1.25 times (AOR 1.25, 95% CI (1.01–1.56), *p* = 0.048). In comparison with adolescents aged 16 years old, those aged 14 years old had a lower likelihood of reporting internalizing behaviors (AOR 0.68, 95% CI (0.44–0.89), *p* = 0.009). Adolescents from aided schools had a 2.09 times increased odds of reporting internalizing behaviors (AOR 2.09, 95% CI (1.30–3.35), *p* = 0.002).

## 4. Discussion

We explored the predictors of behaviors among in-school adolescents aged 14–16 years old. We assessed the determinants of the SDQ domains, including externalizing and internalizing behaviors, using self-reports. In brief, we found that the majority of adolescents scored under the normal category. Borderline scores were the highest in the peer relationship domain (17.8%), followed by the emotional problems domain (7.8%), while in the total difficulties score, they constituted 12.7%. In assessing for abnormal scores, we found that they were higher for conduct problems (8.5%) than in the other domains, and abnormal total difficulties scores reached 5.1% among our in-school participants. In comparison, in a study among adolescents in the Chinese context, Wang et al. found that a higher proportion of the participants (17.9%) had a high total difficulties score [28]. Data from the Indian subcontinent showed that more than a tenth of in-school adolescents reported abnormal scores in the SDQ in a study by Harikrishnan, U. and Sailo, G.L. in Kollam, Kerala [29]. Nair, S. et al. in their study among in-school adolescents in Anand, Gujarat, found that 15% of their participants had an abnormal total difficulties score [30], which was higher than our findings. However, SDQ scores have been found to differ across India in different school settings, as an observed total difficulties score of 8.35% was reported by Shekhawat, R. et al. among adolescents in Jaipur, Rajasthan [31].

The predictors of total difficulties in our study were the age of the participants and the type of school. In our study, younger adolescents aged 14 years old were less likely to have abnormal scores. While among our participants a younger age was protective against an abnormal score, Van Roy, B. et al. found that scores were higher among younger participants in their study [32]. Among Chinese adolescents, abnormal scores were also predicted by the age of the adolescents [20]. Study findings from India and other places have reported higher family engagement and parental involvement are protective factors for adolescent mental health [33]. However, parental involvement decreases with an increase in age, and mental health problems increase with age [34]. This may support the findings of the current study.

We found that the type of school also predicted an abnormal score. In our study, we found that adolescents from aided schools reported higher total difficulty scores than adolescents in private schools. This may be because of the scarcity of availability of school counselors in aided and government schools [35]. Other studies in the Indian context have confirmed similar findings [36,37].

Being male predicted higher externalizing behavior scores. In the literature, gender has been a determinant of both internalizing and externalizing behaviors. Bishop, S. et al., in their study among Nigerian adolescents, reported that being male was associated with aggression [38]. These findings are similar to those reported by Aboobaker, S. et al. [39] in the Indian context and are comparable with the findings of our study. Being female, in our study, predicted internalizing behaviors. Gutman, L. et al. argue that while female adolescents may be less likely to engage in delinquent behavior, participation in such behavior may undergird depression or anxiety [40]. This is determined by various social and biological determinants. Our findings are supported by the literature from Western settings as well [41,42,43].

Among the participants, gender differences were elicited in the SDQ domains. In our study, female participants had greater emotional problems as compared to males. This finding was comparable with those of Nair, S., who also measured that female participants from Gujarat had higher scores in terms of emotional problems compared with male participants [30]. This finding was also confirmed by Puwar, T. et al. [44] and Aboobaker, S. et al. in other settings in India [37]; Wang et al. [40] in China; and Rimal, H.S. and Pokharel, A. in Nepal [45]. Harikrishnan, U. et al. [36] reported that the age of the participants was positively correlated with emotional problems in a study among adolescents in Tezpur, Assam. In comparison, in data from adolescents from Norway, Van Roy et al. also found that girls had a higher total difficulties score in comparison to their male counterparts [32]. Van Roy et al. found that male participants reported higher levels of conduct and peer-related problems. This finding is also comparable with the findings of our study. The prosocial scores, which measure positive attributes, were, however, higher among our female participants, which is similar to the findings of Van Roy et al. 

## 5. Conclusions and Recommendations

In conclusion, the in-school adolescents in this study had lower total difficulties scores in comparison with those from similar studies in India. This may be due to higher access to mental health services in Udupi taluk. Also, private schools with central boards have counsellors in school. The age and gender of the participants and the types of schools emerged as significant predictors of the total difficulties and the externalizing and internalizing behavioral problems among them. To aid adolescents in overcoming adverse behavioral outcomes, policies and interventions need to take a multi-pronged approach. The school setting may prove to be an ideal resource in creating awareness and providing life skills education to equip adolescents with. School-based mental health promotion and prevention with early intervention may be instrumental in promoting and enhancing mental wellbeing and strengthening individuals’ capacities to build resilience.

## Figures and Tables

**Table 1:** Socio-demographic characteristics of participants (*n* = 1441).

Characteristics	Frequency (%)
Area of residence	
Urban	644 (44.7%)
Rural	797 (55.3%)
Sex	
Male	715 (49.6%)
Female	726 (50.4%)
Age (years)	
14	223 (15.5%)
15	536 (37.2%)
16	682 (47.3%)
Type of school	
Government	550 (38.2%)
Aided	346 (24%)
Private	545 (37.8%)

**Table 2:** Self-reported emotional and behavioral problems across SDQ domains (*n* = 1441).

SDQ Domains	Frequency (%)		
	Normal	Borderline	Abnormal
Emotional symptoms	1230 (85.4%)	112 (7.8%)	99 (6.9%)
Conduct problems	1159 (80.4%)	160 (11.1%)	122 (8.5%)
Hyperactivity/inattention	1312 (91%)	81 (5.6%)	48 (3.3%)
Peer relationships	1139 (79%)	256 (17.8%)	46 (3.2%)
Prosocial behaviors	1370 (95.1%)	45 (3.1%)	26 (1.8%)
Total difficulties	1184 (82.2%)	183 (12.7%)	74 (5.1%)

**Table 3:** Gender differences in emotional and behavioral problems on assessing using independent *t*-tests.

Domains	Male	Female	t	*p*-Value	Cohen’s d
	Mean (SD)	Mean (SD)			
Emotional problems	3.01 (1.98)	3.51 (2.07)	4.65	<0.0001 **	0.25
Conduct problems	2.39 (1.58)	2.22 (1.43)	2.28	0.023 *	0.11
Hyperactivity	3.20 (1.79)	2.90 (1.83)	3.06	0.002 *	0.17
Peer relationships	2.52 (1.48)	2.32 (1.45)	2.53	0.011 *	0.14
Prosocial behaviors	8.21 (1.56)	8.59 (1.45)	4.88	<0.0001 **	0.25
Externalizing behaviors	5.59 (2.80)	5.12 (2.75)	3.24	0.001 *	0.16
Internalizing behaviors	5.52 (2.87)	5.83 (2.79)	2.02	0.043 *	0.11
Total SDQ	11.13 (4.95)	10.95 (4.78)	0.67	0.50	0.04

*p* < 0.0001 ** *p* < 0.05 *.

**Table 4:** Univariate and multivariate analysis of predictors of SDQ domains.

**SDQ—Total Difficulties**		
**Variables ^@^**	**AOR 95% CI**	***p*-Value**
Age (years)		
14	0.57 (0.36–0.89)	0.014
15	0.89 (0.67–1.19)	0.45
16	Reference	
Type of school		
Government	1.01 (0.73–1.39)	0.97
Aided	1.65 (1.18–2.31)	0.004
Private	Reference	
**Externalizing Behaviors**		
**Variables ^#^**	**AOR 95% CI**	***p*-Value**
Gender		
Male	1.49 (1.09–2.04)	0.013
Female	Reference	
**Internalizing Behaviors**		
**Variables ^$^**	**AOR 95% CI**	***p*-Value**
Gender		
Female	1.25 (1.01–1.56)	0.048
Male	Reference	
Age (years)		
14	0.68 (0.44–0.89)	0.009
15	0.79 (0.44–0.89)	0.069
16	Reference	
Type of school		
Government	1.47 (0.81–2.69)	0.200
Aided	2.08 (1.30–3.34)	0.002
Private	Reference	

^@^ Adjusted for age of the participants and the type of school. ^#^ Adjusted for area of residence, gender, type of school, and the age of the participant. ^$^ Adjusted for area of residence, gender, type of school, socioeconomic status, and the age of the participant.

## Data Availability

They will be provided on request.
